# Supplementary figures and images for: GLI3 resides at the intersection of hedgehog and androgen action to promote male sex differentiation
Source: PLoS Genet. 2020 Jun 4;16(6):e1008810. doi: 10.1371/journal.pgen.1008810 (PMC7297385; doi:10.1371/journal.pgen.1008810)

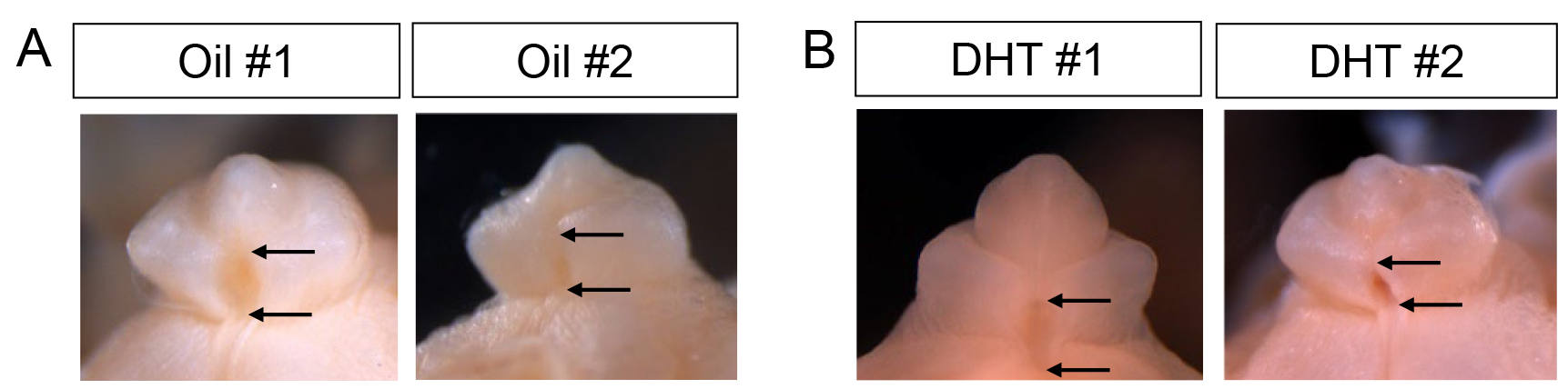

Supplement: S1 Fig — External genitalia of Gli3XtJ males harvested from pregnant dams that were treated daily with sesame oil (A) (n = 7) or DHT (1 mg/kg body weight) (B) (n = 8) beginning at E12.5. Arrows indicate ectopic opening of hypospadiac urethra. Embryos were harvested at E16.5 or E17.5 and imaged using whole-mount light microscopy. (TIF) [file pgen.1008810.s001.tif]

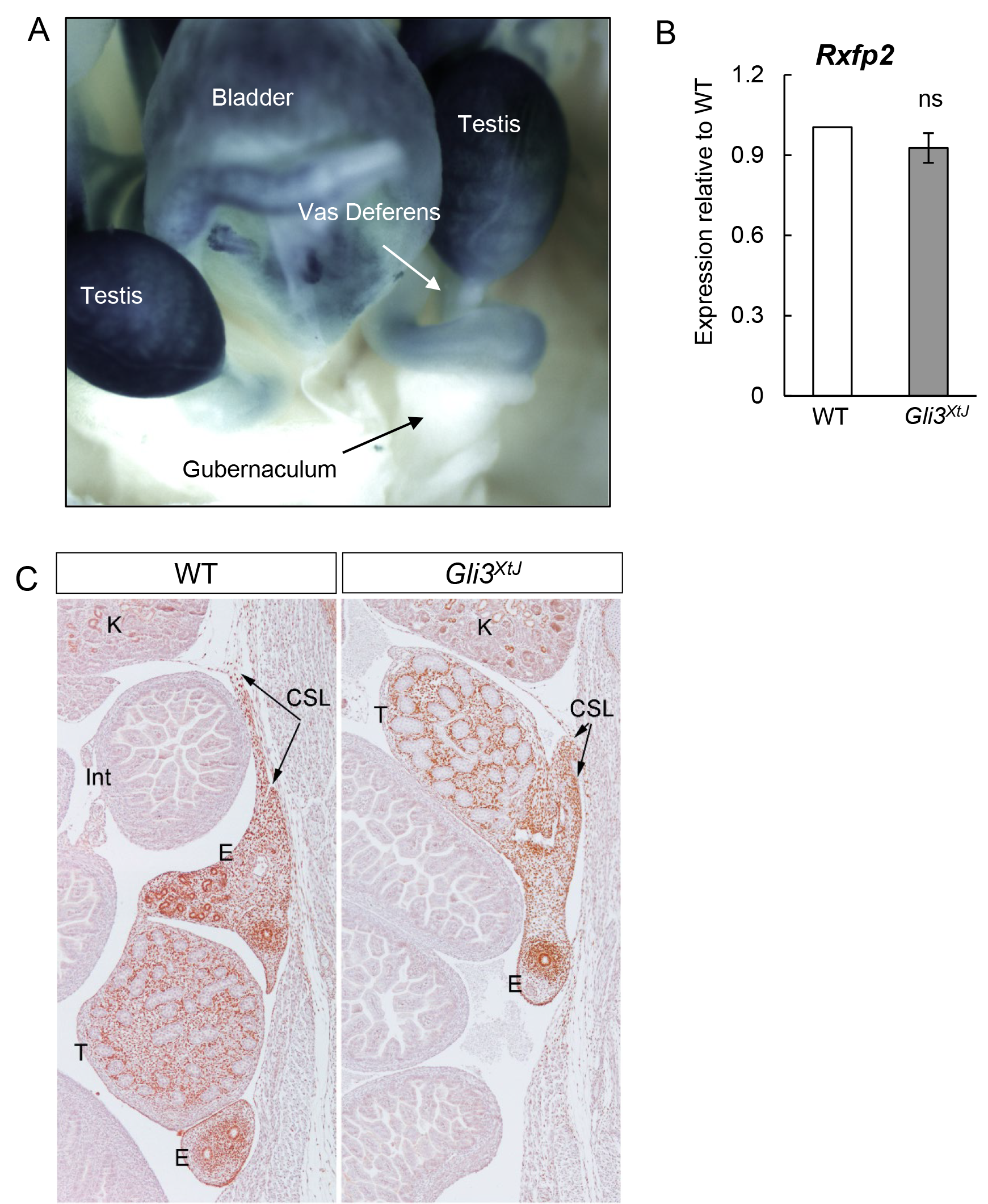

Supplement: S2 Fig — A) β-galactosidase (blue) expression in urogenital tracts of a Gli1-LacZ P0 male. The white arrow highlights positive LacZ stain in the vas deferens and the black arrow points out the lack of staining in the gubernaculum B) Expression of INSL3 receptor, Rxfp2, is unaffected in Gli3XtJ gubernaculum at E16.5 compared to the wild type. Results are normalized to wild type control and are represented as mean ± SEM from three biological replicates. Student’s t-test, ns: non-significant. C) IHC with anti-androgen receptor (AR) antibody shows similar expression pattern in wild type and Gli3XtJ mutant embryos. Arrows denote cranial suspensory ligaments (CSL); epididymis (E), intestine (Int), kidney (K), testis (T). (TIF) [file pgen.1008810.s002.tif]

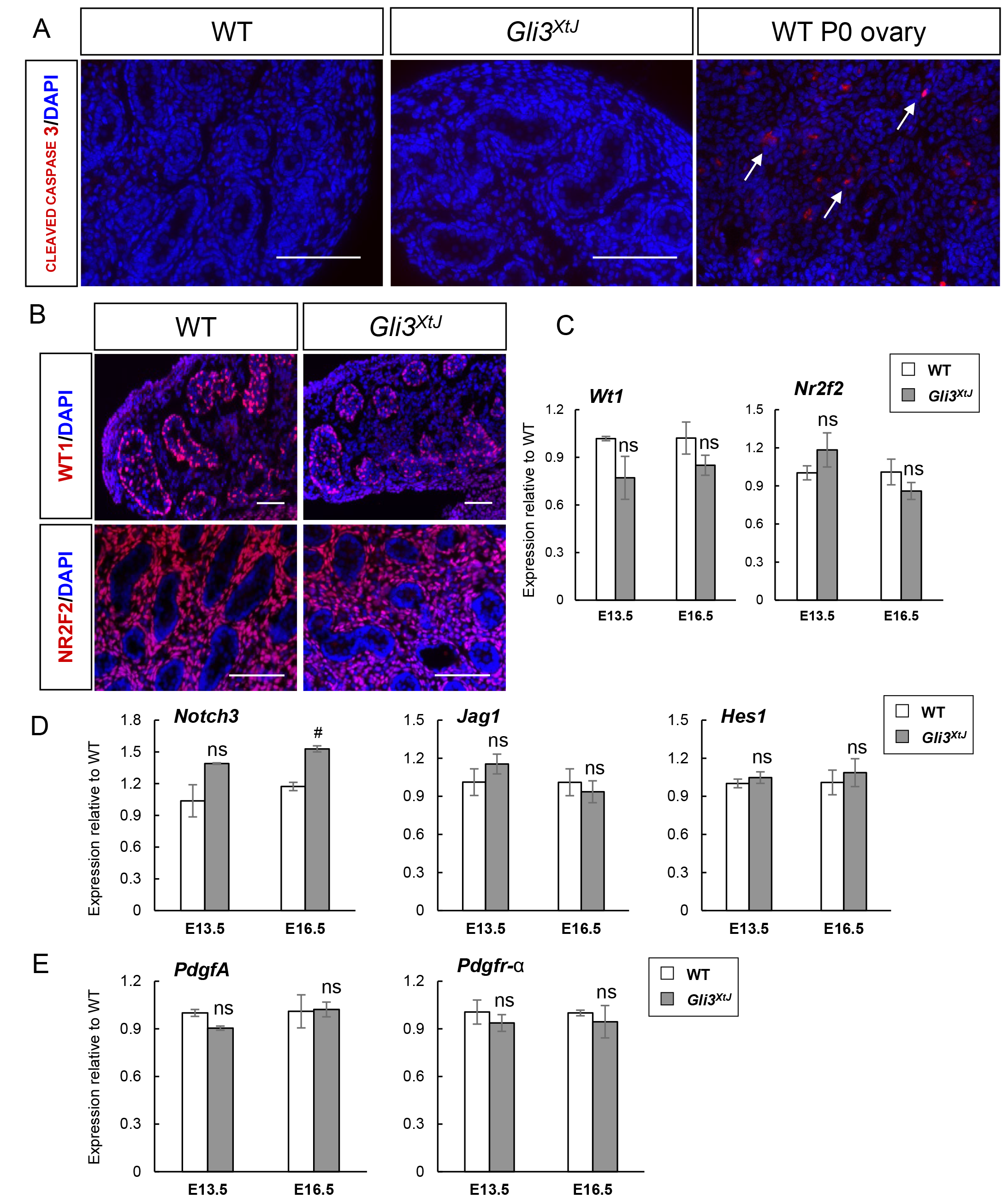

Supplement: S3 Fig — A) Expression of cleaved caspase 3 with DAPI nuclear counterstain at E16.5 in wild type and Gli3XtJ testes. P0 ovary is included as a positive control, arrows highlight positive staining for cleaved caspase 3. B,C) Expression levels of Wt1 and Nr2f2 were analyzed by IHC (B, Scale bar: 100 μm) and RT-qPCR (C). D, E) Transcript levels of Notch signaling pathway genes (D) and PDGF signaling pathway genes (E) were analyzed by RT-qPCR. Results are normalized to wild type controls at each age and are represented as mean ± SEM from three biological replicates. Student’s t-test, #: p<0.1, ns: non-significant. (TIF) [file pgen.1008810.s003.tif]

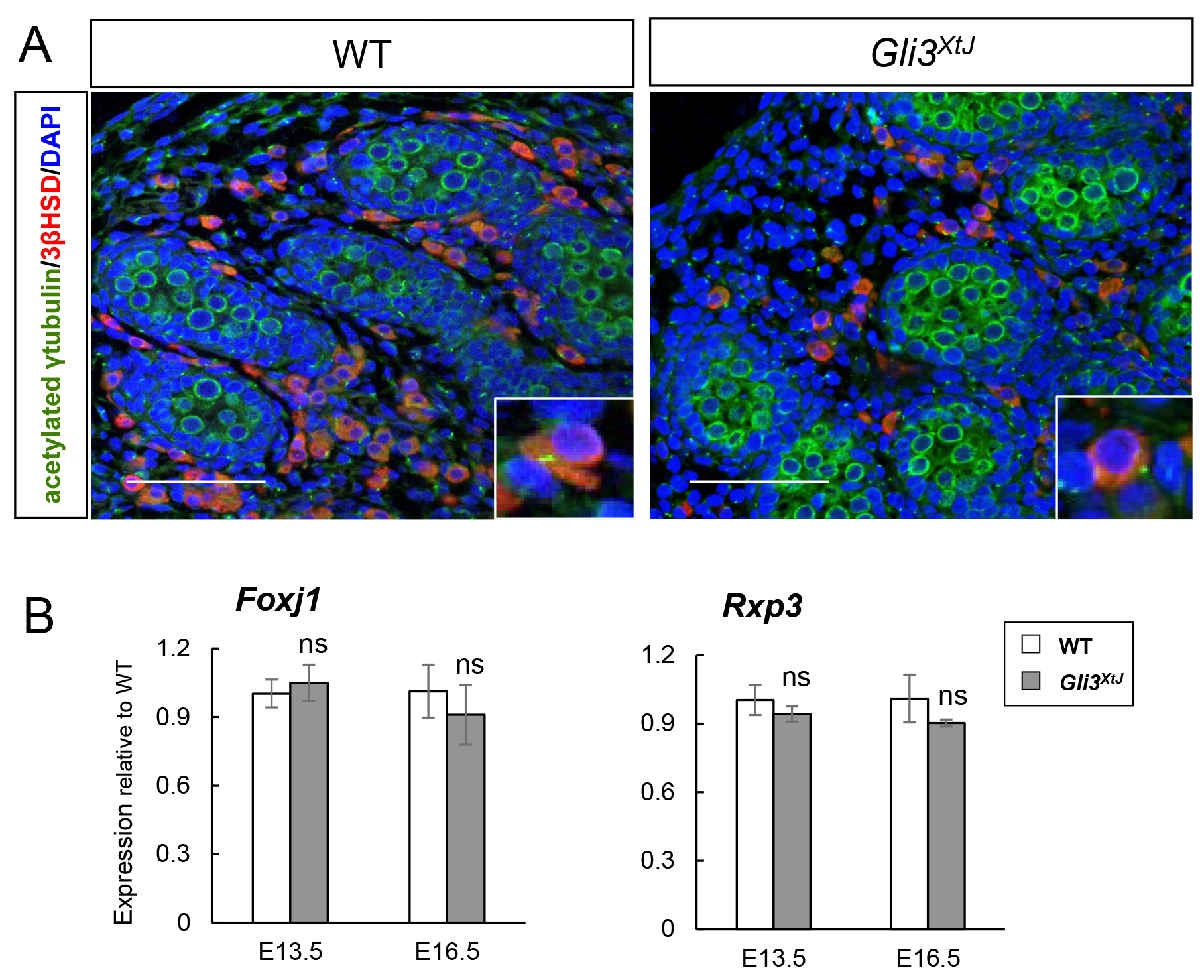

Supplement: S4 Fig — A) Representative images of acetylated γ-tubulin (green) and 3βHSD immunostained (red, counterstained with DAPI) wild type and Gli3XtJ testes at E16.5 showing similar appearance of cilia. Scale bar: 100 μm. B) Transcript levels of genes involved in ciliary biogenesis, Foxj1 and Rxp3 are unaffected in Gli3XtJ testes at E13.5 and E16.5 compared to wild type. Results are reported as compared to wild type controls at each age and represented as mean ± SEM from three biological replicates. Student’s t-test, ns: non-significant. (TIF) [file pgen.1008810.s004.tif]

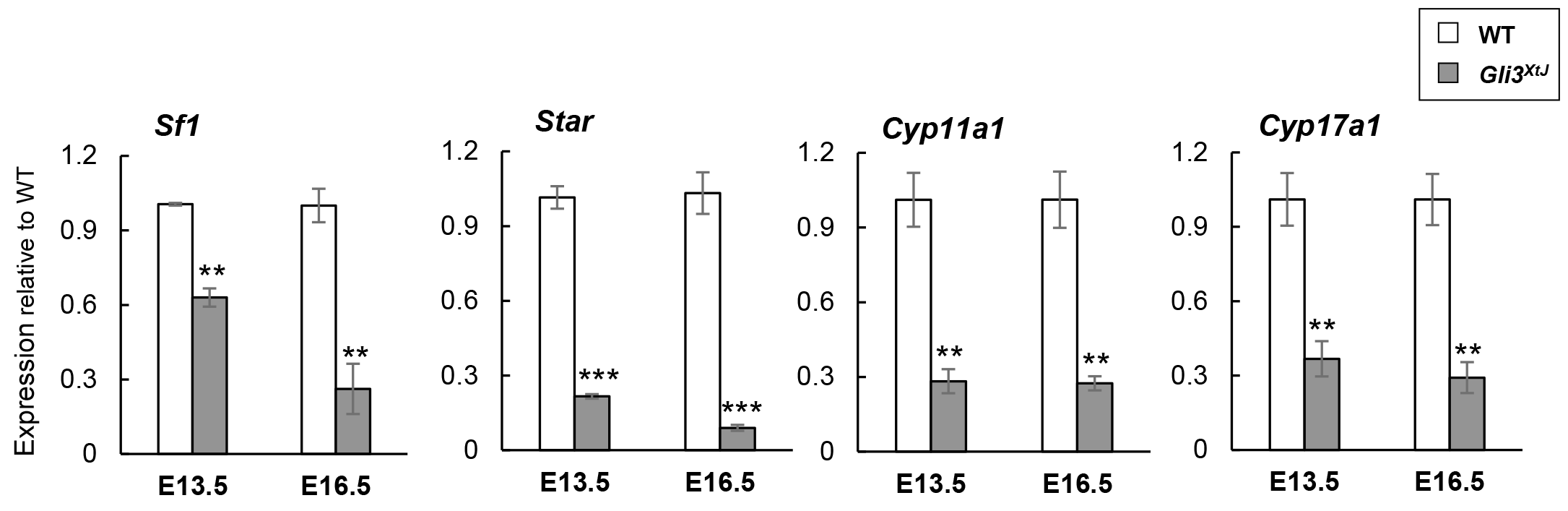

Supplement: S5 Fig — RT-qPCR comparing wild type and Gli3XtJ testes mRNA expression of steroidogenic genes at E13.5 and E16.5 normalized to 36b4 (Rplpo) and expressed as fold change from wild type testes ± SEM, from n = 3–4 biological replicates. Student’s t-test, **: p<0.01, ***: p<0.001. (TIF) [file pgen.1008810.s005.tif]

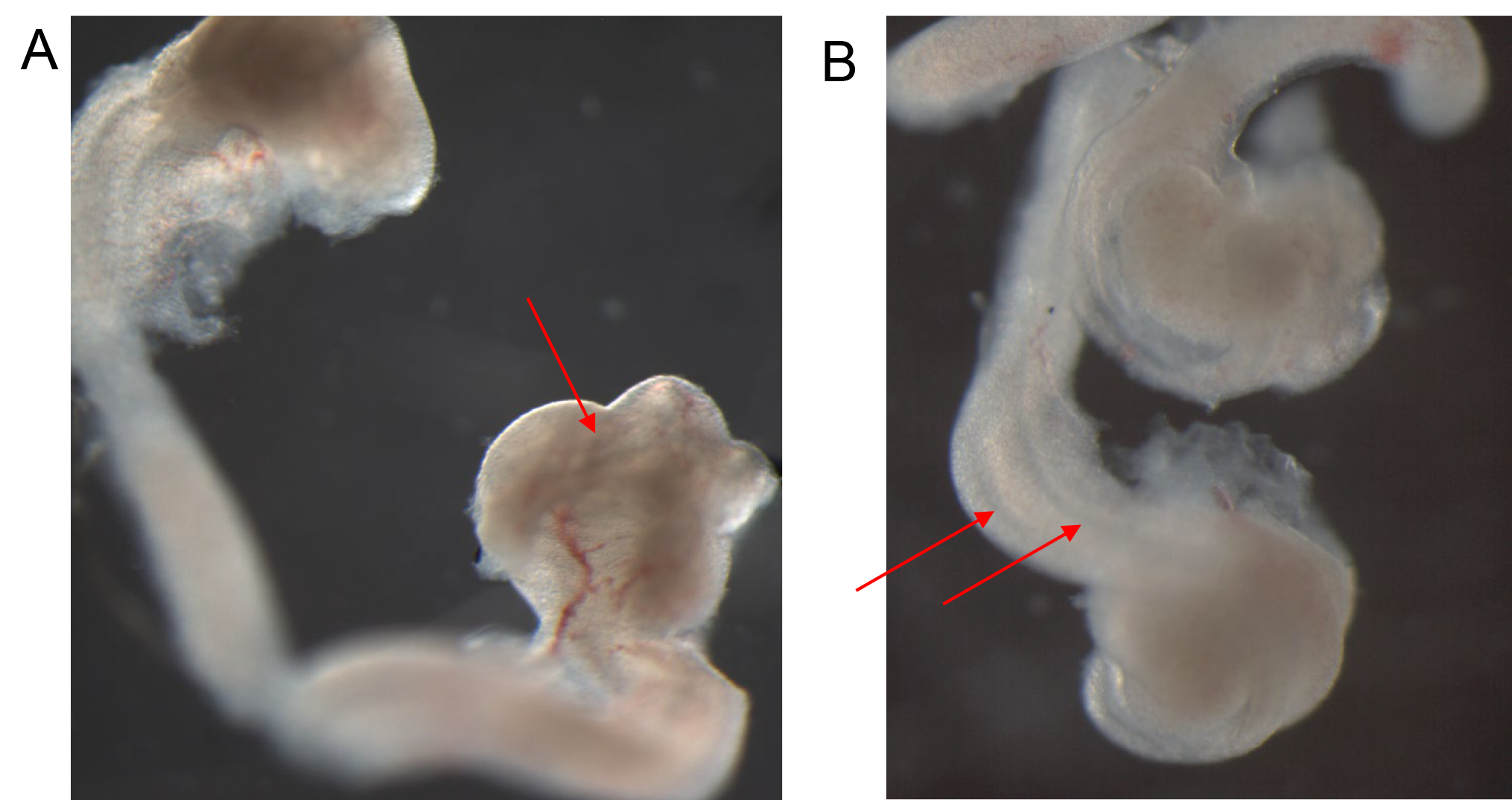

Supplement: S6 Fig — A,B) Pregnant dams (n = 5) were treated daily with sesame oil or DHT (1 mg/kg body weight), starting on E12.5 and harvested at E16.5. Female reproductive tracts were examined under phase contrast microscopy. Arrows indicate epididymis head (A) and the presence of both the Müllerian and Wolffian ducts (B). (TIF) [file pgen.1008810.s006.tif]
