# Supplementary material for: GLI3 resides at the intersection of hedgehog and androgen action to promote male sex differentiation
Source: PLoS Genet. 2020 Jun 4;16(6):e1008810. doi: 10.1371/journal.pgen.1008810 (PMC7297385; doi:10.1371/journal.pgen.1008810)
Supplement: S1 Table — (DOCX) [file pgen.1008810.s007.docx]

**S1 Table. RT-qPCR Primers**

| Gene | Forward 5'-3' | Reverse 5'-3' |
| --- | --- | --- |
| *Dhh* | ACCCCGACATAATCTTCAAGGA | GTTCACCCGCTCTTTGCAA |
| *Ptch1* | CTAGCAATAGGGACCGCTCA | GTCTCAGGGTAGCTCTCATAG |
| *Gli1* | TGTGGCGAATAGACAGAGGT | TGCCAGATATGCTTCAGCCA |
| *Gli2* | AGCCTTCACCCACCTTCTTG | TGGGCGCAGGCCCTCAGC |
| *Gli3* | CCGTTCAAAGCCCAGTACAT | TGAGTAGGCTTTTGTGCAACC |
| *Sf1* | TCTCTAACCGCACCATCAAG | TCGACAATGGAGATAAAGGTC |
| *Star* | GAGTGGTGTCATCAGAGCTGAAC | TGAGTTTAGTCTTGGAGGGACTTCC |
| *Cyp11a1* | CGCATAAAGCAGCAAAATTC | ATGCGCTCCCCAAATATAAC |
| *Cyp17a1* | AAGCATATCCTTGTCACGGTGG | ACGGTGTTCGACTGAAGCCTAC |
| *Insl3* | TGGCTAGAGCAGAGACATC | CCTGTGGTCCTTGCTTAC |
| *Rxfp2* | CCATGGGAATGTCAATAAAGTG | TCTGCAGTAACAGTGCTGTGG |
| *Sox9* | AGTACCCGCATCTGCACAAC | TACTTGTAATCGGGGTGGTCT |
| *36b4* | CGACCTGGAAGTCCAACTAC | ATCTGCTGCATCTGCTTG |
| *Foxj1* | CTCCTATGCCACTCTCATCTGC | GACAGGTTGTGGCGGATGGAAT |
| *Rxp3* | CGCAAAAAGCCTAGAAGGTTGGC | GCCAGGTGATTGAGCGATGTGT |
| *Nr2f2* | TGCGGAGGAACCTGAGCTAC | CTGTACAGCTTCCCGTCTCAT |
| *Wt1* | CAAGGACTGCGAGAGAAGGTTT | TGGTGTGGGTCTTCAGATGGT |
| *Notch3* | AGATCAATGAGTGTGCATCC | GCAGACTCCATGACTACAGG |
| *Jag1* | TGACATGGATAAACACCAGCA | GCAGCCCACTGTCTGCTATAC |
| *Hes1* | TACCCCAGCCAGTGTCAACA | CCTTCGCCTCTTCTCCATGA |
| *PdgfA* | TGAAAGAGGTCCAGGTGAGG | CACGGAGGAGAACAAAGACC |
| *Pdgfr-α* | TCCATGCTAGACTCAGAAGTCA | TCCCGGTGGACACAATTTTTC |
| *Sry* | TGCAGCTCTACTCCAGTCTTG | AGATCTTGATTTTTAGTGTTC |
