# Supplementary material for: GLI3 resides at the intersection of hedgehog and androgen action to promote male sex differentiation
Source: PLoS Genet. 2020 Jun 4;16(6):e1008810. doi: 10.1371/journal.pgen.1008810 (PMC7297385; doi:10.1371/journal.pgen.1008810)
Supplement: S2 Table — (DOCX) [file pgen.1008810.s008.docx]

**S2 Table. Antibodies**

| **Antibody** | **Source** | **Catalogue #** |
| --- | --- | --- |
| Rabbit anti Androgen Receptor | Santa Cruz Biotechnology | sc-816 |
| Rabbit anti 3βHSD | TransGenic Inc | KO607 |
| Mouse anti NR2F2 | R&D Systems | PP-H7147 |
| Mouse anti WT1 | Dako | Clone 6F-H2 |
| Mouse anti γ-acetylated tubulin | Sigma-Aldrich | T5326 |
| Mouse anti TRA98 | Cosmo Bio Co Ltd | 73003 |
| Goat anti AMH | Santa Cruz Biotechnology | sc-6886 |
| Rabbit anti Laminin | Sigma-Aldrich | L9393 |
| Chicken anti β-galactosidase | Abcam | Ab9361 |
| Rabbit anti Cleaved Caspase 3 | Cell Signaling Technology | 9661 |
| Immuno-pure goat anti-rabbit IgG Rhodamin conjugated | Pierce | 31670 |
| Immuno-pure rabbit anti-goat IgG Rhodamin conjugated | Pierce | 31650 |
| Alexa Fluor 546 donkey anti-rabbit IgG (H+L) | Invitrogen | A-10040 |
| Alexa Fluor 568 donkey anti-rabbit IgG (H+L) | Invitrogen | A-10042 |
| Alexa Fluor 488 donkey anti-mouse IgG (H+L) | Invitrogen | A-21202 |
| Alexa Fluor 647 donkey anti-chicken IgY (H+L) | Jackson ImmunoResearch | 703-605-155 |
| Alexa Fluor 546 goat anti-chicken IgY (H+L) | Invitrogen | A-11040 |
