# Supplementary material for: GLI3 resides at the intersection of hedgehog and androgen action to promote male sex differentiation
Source: PLoS Genet. 2020 Jun 4;16(6):e1008810. doi: 10.1371/journal.pgen.1008810 (PMC7297385; doi:10.1371/journal.pgen.1008810)
Supplement: S3 Table — (DOCX) [file pgen.1008810.s009.docx]

**S3 Table. Gblock sequences for Copy Number PCR**

Copy Number PCR was performed using Gblock composite sequences listed below (IDT Technologies). For completeness, all sequences are shown; the primers for each gene of interest used in Fig 7 are highlighted in the tables below each relevant sequence.

**Gblock 1 composite sequence**:

| ACCCCGACATAATCTTCAAGGATGAGGAGAACAGCGGCGCAGACCGCCTGATGACAGAGCGTTGCAAAGAGCGGGTGAACACCAACCAACTATGGCCCTGGCCACTGTGCCCAGCAGGTCTCCTATCCTGATCCCACCCCAGAAAACTGGGGTGAGTTCCCTTCTCATGCTGGGGTGTACCCTAACACTGTGGAGGACTGCCTACATATCAAAGCCATCAAGACAGAGAGCTCCGGGCTTTGTCAGTCCAGCCCCGGGGCCCAGTCATCCTGCAGCAGCCCGTTCAAAGCCCAGTACATGTTGGTAGTGCATATGAGAAGACACACTGGGGAGAAGCCTCACAAATGTACATTTGAAGGTTGCACAAAAGCCTACTCACTTGTGGAATGCCTTGTGATTGGAGTTGGCACCATGTCAGGCGTCCGCCAGCTGGAGATCATGTGCTGCTTCGGCTCGACCTGGAAGTCCAACTACTTCCTCAAGATCATCCAACTTTTGGATGATTATCCAAAATGCTTCATTGTGGGAGCAGACAACGTGGGCTCCAAGCAGATGCAGCAGATGGCAAGTTTTTGGTTGTGGGTCTCCTCATATTTGGGGCCTTCGCTGTGGGATTAAAGGCAGCTAATCTCGAGACCAACGTGGAGGAGCTGTGGGTGGAAGTTGGTGGACGAGTGAGTCGAGAATTAAATTATACCCGTCAGAAGATAGGAGAAGAGGTTGTGCTCATCACCTTCAGCTGCCACTTCTATGACTTCTTCAACCAGGCTGAGTGGGAGCGTAGCTTCCGGGACTATGTGCTATGCCAAGCCA |
| --- |
| **Gblock 1 primer sequences**:   \| **Gene** \| **Forward 5'-3'** \| **Reverse 5'-3'** \| \| --- \| --- \| --- \| \| *Dhh* \| ACCCCGACATAATCTTCAAGGA \| GTTCACCCGCTCTTTGCAA \| \| *Gli1* \| ACCAACCAACTATGGCCCTG \| TAGGGTACACCCCAGCATGA \| \| *Gli2* \| ACACTGTGGAGGACTGCCTA \| GCTGCTGCAGGATGACTG \| \| *Gli3* \| CCGTTCAAAGCCCAGTACAT \| TGAGTAGGCTTTTGTGCAACC \| \| *HMGCoA* \| CTTGTGGAATGCCTTGTGATTG \| AGCCGAAGCAGCACATGAT \| \| *36B4* \| CGACCTGGAAGTCCAACTAC \| ATCTGCTGCATCTGCTTG \| \| *Ptch1* \| GGCAAGTTTTTGGTTGTGGGTC \| CCTCTTCTCCTATCTTCTGACGGG \| \| *Smo* \| TTGTGCTCATCACCTTCAGC \| TGGCTTGGCATAGCACATAG \| |

**Gblock 2 composite sequence**:

GGATGCGTCGATACTCTTCTCATGCGAGGGTCCCAACCCGGAGCGGTTCCTTGTGCCCCCCTGGGTGGCCTATCACCAGTATTATCAGAGGCCCATTGGGGTCCTGTTTAAGAGTTCAGATGCCTGGAAGGAGTGGTGTCATCAGAGCTGAACACGGCCCCACCTGCATGGTGCTTCATCCACTGGCTGGAAGTCCCTCCAAGACTAAACTCAAGAGCCTGCCAGGGCATCTCTGTTGTCATCCACACTGCTGCTGTCATTGATGTCACAGGTGTCATTCCCAGGCAGACCATCCTAGATGTCAATCTGAAAGGTACCCAGAACCTATTGGAGGCCTGTGCCCAAGCAAGCGTGCCAAAGGTTTGTGCGAGAGTCTGGCGATCCTGCTGCCGCTCTTTGGAGTCCATGTGAGCCTCATCGAGTGTGGGGCAGTGCACACAGCCTTCTATGAAAAGCTGGTGAGGTGCATGGTCTTTAAGGAGCTGGAGGTGGCTGACCAGATGACACTGCTGCAGAACTGTTGGAGCGAGCTGCTGGTGTTGGACCACATCTACCGCCAGGTCCAGTACGGCAAGGAAGACAGAAGCATATCCTTGTCACGGTGGGAGACATCTTTGGGGCAGGCATAGAGACAACTAGCTCTGTGCTGAACTGGATCCTGGCTTTCCTGGTGCACAATCCTGAGGTGAAGAGGAAGATCCAAAAGGAGATTGACCAGTATGTAGGCTTCAGTCGAACACCGTCATCCTTCCACAGTTCTAGCTCCAGACTGGGACTGCTGACACCCCACACCTTGTGGCTGACCATCTCCAGGGTCACTGTACATTAATCTCACACCAGGCGGTGCGTCCTTAATTGCTCCCCACCTCCTGGCTATGTCATTGCAACAATCCAGTTTGCCTTGGAGGATCCCTAGTTTATTCATAGATCGCAGTAAGCAAGGACCACAGGCCCTGTCCCCAAGAAGCCCCATCATGACCTGTGCCCAC

**Gblock 2 primer sequence**:

| **Gene** | **Forward 5'-3'** | **Reverse 5'-3'** |
| --- | --- | --- |
| *Cyp11a1* | GGATGCGTCGATACTCTTCTC | CTTCCAGGCATCTGAACTCTTA |
| *Star* | GAGTGGTGTCATCAGAGCTGAAC | TGAGTTTAGTCTTGGAGGGACTTCC |
| *3βHSD* | AGAGCCTGCCAGGGCATCTCTGTT | TGGCACGCTTGCTTGGGCACAGGC |
| *17βHSD* | AAGGTTTGTGCGAGAGTCTG | CACCAGCTTTTCATAGAAGGC |
| *Sf1* | AGGTGCATGGTCTTTAAGGAG | CTGTCTTCCTTGCCGTACTG |
| *Cyp17a1* | AAGCATATCCTTGTCACGGTGG | ACGGTGTTCGACTGAAGCCTAC |
| *6βHSD* | CATCCTTCCACAGTTCTAGC | TGGTGTGAGATTAATGTACA |
| *Insl3* | GGCGGTGCGTCCTTAATTG | GTGGGCACAGGTCATGATG |

**Gblock 3 composite sequence**:

AGGAAGCTGGCAGACCAGTACCCGCATCTGCACAACGCGGAGCTCAGCAAGACTCTGGGCAAGCTCTGGAGGCTGCTGAATGAGAGCGAGAAGACCGTTCAAAGCCCAGTACATGTTGGTAGTGCATATGAGAAGACACACTGGGGAGAAGCCTCACAAATGTACATTTGAAGGTTGCACAAAAGCCTACTCAACACTGTGGAGGACTGCCTACATATCAAAGCCATCAAGACAGAGAGCTCCGGGCTTTGTCAGTCCAGCCCCGGGGCCCAGTCATCCTGCAGCAGCAGAGCCTGCCAGGGCATCTCTGTTGTCATCCACACTGCTGCTGTCATTGATGTCACAGGTGTCATTCCCAGGCAGACCATCCTAGATGTCAATCTGAAAGGTACCCAGAACCTATTGGAGGCCTGTGCCCAAGCAAGCGTGCCAGTCCTTGTCCAGTGTATTGTAAAGAGCCACCTCGGAGCCTGGAACAGCAGCTACAGGAGCATAGGCTCCAACAGAAGCGACTCTTCCTCCAGAAGCAGTCTCAGCTGCAAGCATATTTTAATCAGATGCAGATAGCAGAGAGGTGCATGGTCTTTAAGGAGCTGGAGGTGGCTGACCAGATGACACTGCTGCAGAACTGTTGGAGCGAGCTGCTGGTGTTGGACCACATCTACCGCCAGGTCCAGTACGGCAAGGAAGACAGACAGATTGCCACATTAGCCCAGGTATCCATGCCAGCAGCTCATGCAACATCATCTGCTCCCACTGTAACCTTAGTGCAGCTGCCCAATGGGCAGACAGTCCAGGTCCATGGCGTTATCCAGGCGGCCCAGCCATCAGTTATCCAGTCTCTTGACGGAAGGGCACCACCAGGAGTGGAGCCTGCGGCTTAATTTGACTCAACACGGGAAACCTCACCCGGCCCGGACACGGACAGGATTGACAGATTGATAGCTCTTTCTCGATTCCGTGGGTGGTGGTGC

**Gblock 3 primer sequence**:

| **Gene** | **Forward 5'-3'** | **Reverse 5'-3'** |
| --- | --- | --- |
| *Sox9* | AGGAAGCTGGCAGACCAGTA | TCTTCTCGCTCTCATTCAGC |
| *Gli3* | CCGTTCAAAGCCCAGTACAT | TGAGTAGGCTTTTGTGCAACC |
| *Gli2* | ACACTGTGGAGGACTGCCTA | GCTGCTGCAGGATGACTG |
| *3βHSD* | AGAGCCTGCCAGGGCATCTCTGTT | TGGCACGCTTGCTTGGGCACAGGC |
| *Sik2* | GTCCTTGTCCAGTGTATTGTAA | CTCTGCTATCTGCATCTGATT |
| *Sf1* | AGGTGCATGGTCTTTAAGGAG | CTGTCTTCCTTGCCGTACTG |
| *Creb* | ACAGATTGCCACATTAGCCC | GAGACTGGATAACTGATGGCTG |
| *18s* | TTGACGGAAGGGCACCACCAG | GCACCACCACCCACGGAATCG |
