# Supplementary material for: GLI3 resides at the intersection of hedgehog and androgen action to promote male sex differentiation
Source: PLoS Genet. 2020 Jun 4;16(6):e1008810. doi: 10.1371/journal.pgen.1008810 (PMC7297385; doi:10.1371/journal.pgen.1008810)
Supplement: S1 Methods — (DOCX) [file pgen.1008810.s010.docx]

**S1 METHODS**

**AR Immunohistochemistry**

Embryos were fixed in 4% paraformaldehyde overnight at 4°C, processed and embedded in paraffin. Paraffin blocks were sectioned at 7 μm thickness and sections were stained with androgen receptor (AR) antibody (sc-816, Santa Cruz Biotechnology, Santa Cruz, CA). Detection was performed using a Vectastain ABC (avidin–biotin–peroxidase) kit (Vector Laboratories) as recommended. The color was developed with diaminobenzidine (DAB) as chromogen. Samples were counterstained with Harris Hematoxylin. Stained slides were examined with a Carl Zeiss Axio A1 Microscope, and images were captured by an AxioCam MRc5 CCD camera.

**Whole Mount LacZ staining**

Embryos were fixed in 4% paraformaldehyde for 40 min at 4°C and stained with X-gal substrate (Invitrogen). Once the color developed, the embryos were rinsed and stored in 4% paraformaldehyde at 4°C. Images were captured on a Leica M165FC stereo microscope and processed with Adobe Photoshop.
