# Supplementary material for: GLI3 resides at the intersection of hedgehog and androgen action to promote male sex differentiation
Source: PLoS Genet. 2020 Jun 4;16(6):e1008810. doi: 10.1371/journal.pgen.1008810 (PMC7297385; doi:10.1371/journal.pgen.1008810)
Supplement: Attachment — Submitted filename: 2020 3-24 Plos Genetics Response to Reviewers.docx [file pgen.1008810.s011.docx]

Thank you to all reviewers and associate editors for your consideration with our manuscript, we truly value this process and strongly believe that our reviewers have helped us to improve the presentation of our complicated, but important findings. In response to reviewer comments, we are excited to present new text and model figure that we believe improves the clarity of our results. We sincerely hope that you agree. We have responded to each comment below in blue text. Page/line references match the updated manuscript.

**Reviewer #1**

This manuscript explores how Hedgehog-GLI signaling and androgens interact to drive male urogenital development. In a series of carefully designed functional experiments the authors reveal the crucial role of GLI3 by using Gli3XtJ mutant mice. These mice have a range of phenotypes that affect the male reproductive tract including cryptorchidism and hypospadias. These mice showed lower levels of INSL3 (associated with testis decent) and testosterone. Testis development was otherwise normal in these mice but genes associated with androgen and INSL3 and the DHH pathway were disrupted. This suggests a failure of fetal Leydig cell differentiation and maintenance.

The study clearly shows that these mice have a reduction in the number of fetal Leydig cells which are the source of these testicular hormones and that their ability to function diminished over time. In an effort to separate out the effects of hormones and GLI3 they supplemented these mice with androgens. They show that this partially rescued testis decent but not the hypospadias. However, by adding a GLI3 activator into Gli3XtJ mutant mice testes they restored expression of the Hedgehog pathway and steroidogenic genes. This suggests that fetal Leydig cell function requires activated GLI3 for expression of genes critical to INSL3 and androgen synthesis.

My only real quibble is with data presented in Fig 6B showing a significant decrease in transcript levels of Sf1, Star, CYP11a1 and Cyp17a1. The authors suggest that this may be a real decrease in gene expression or due to fewer fetal Leydig cells or both. If expression levels were adjusted for Leydig cell number then raw values (without adjustment for cell number) showed a more severe impact on expression. The authors should conclude that this therefore means the reduction in expression is largely due to fewer Leydig cells. If this is correct it should be stated as such.

1. This is an important observation. The decrease in transcript levels for steroidogenic factors is largely impacted by the reduced fetal Leydig cell number; however, we observed another 25-40% decrease after values were normalized to fetal Leydig cell number. We clarified our description of results to reflect this on page 12, starting Line 265. This decrease in fetal Leydig cell function is supported by the rescue in transcript values that we observed upon transfection of the Gli3A plasmid (Fig. 7).

The overall message from this study is that appropriate expression of GLI3 is critical for the maintenance of hedgehog signalling and fetal Leydig cell identity. This allows us to build a new paradigm to understand male development. In the developing fetal testis, DHH signals likely initiate Smoothened activity and GLI3 which, in turn, stimulates Insl3, Sf1 and steroid pathway genes which lead to the synthesis of INSL3 and androgens within fetal Leydig cells. This ultimately leads to appropriate male urogenital development.

This is a well written and carefully conducted study that brings new insights to our understanding of male reproductive development. The rising incidence of male urogenital tract defects is discussed briefly. However, in the light of these findings I would like to see a more slightly more detailed discussion of how endocrine disruptors and hedgehog morphogens may impact on male urogenital development.

2. We truly appreciate the kind words and supportive review. We are intrigued by the rise in male urogenital defects, especially within developed countries. As suggested, we briefly expanded the discussion related to how sex differentiation might be impacted by developmental exposures or events (page 13, starting Line 294).

**Reviewer #2**

The paper by Kothandapani et al describes the male reproductive phenotype of GLI3 mutant mice. Since the phenotypes were consistent with a loss of androgen production in this model they supplemented back DHT and observed that some, but not all phenotypes were rescued. This important observation shows the independent impacts of a loss of GLI3 on testis biology as distinct from its role in the development of the external genitalia and anogenital spacing.

This is a well written paper with well designed experiments and a thorough investigation of the role of Gli3 in the male repro tract. Some of the pictures were low-res [no doubt due to pdf conversation] which detracted from some of the beautiful whole mount images.

The only question I was left wondering was if you had looked at downstream HH targets in the GT by qPCR to confirm your hypothesis there?

3. This is a very interesting question, especially considering evidence that shows the importance of both androgens and HH signaling in prenatal genital tubercle development. Evidence suggests that androgen signaling is upstream of the HH pathway at both pre- and postnatal stages of GT development (1). In addition, loss of *Shh* or downstream mediators *Gli2* and *Gli3* each cause hypospadias (2,3,4). It is currently not known whether exogenous androgens could alleviate or bypass these HH-mediated defects. Our studies with the *Gli3^XtJ^* mutant mouse that were subject to exogenous DHT indicate that this would not be the case. Similar to human *Gli3* mutant patients, we observed significant variability in this phenotype that we attributed to unpredictable exposure to androgens. Because our animals did have some circulating androgens, albeit very low, we acknowledge that it is possible that low levels of androgens may be sufficient to promote sufficient HH pathway genes in the face of the *Gli3^XtJ^* mutation. Because supplementation with DHT failed to alleviate those with the phenotype however, we conclude that the *Gli3^XtJ^* mutation is sufficient to impede proper development. Thus, while we agree that it would be interesting to evaluate HH targets in the GT of *Gli3^XtJ^* mutant males, it appears that loss of *Gli3* is sufficient. To address this interesting question, we have expanded our discussion starting on page 15, Line 335.
(1) Zheng Z, Armfield BA, Cohn MJ. Timing of androgen receptor disruption and estrogen exposure underlies a spectrum of congenital penile anomalies. Proc Natl Acad Sci U S A. 2015;112(52):E7194-203.
(2) Seifert AW, Bouldin CM, Choi KS, Harfe BD, Cohn MJ. Multiphasic and tissue-specific roles of sonic hedgehog in cloacal septation and external genitalia development. Development. 2009;136(23):3949-57.
(3) Miyagawa S, Matsumaru D, Murashima A, Omori A, Satoh Y, Haraguchi R, et al. The role of sonic hedgehog-Gli2 pathway in the masculinization of external genitalia. Endocrinology. 2011;152(7):2894-903.
(4) He F, Akbari P, Mo R, Zhang JJ, Hui CC, Kim PC, et al. Adult Gli2+/-;Gli3Delta699/+ Male and Female Mice Display a Spectrum of Genital Malformation. PLoS One. 2016;11(11):e0165958.

Minor corrections:

Line 285 – ‘urethral tube defects associated with hypospadias’ – wording is strange here, urethral plate development was not examined, better to just say ‘caused hypospadias’.

4. Edited as suggested.

Line 301 – ‘reproductive portion of the urogenital tract and the anorectal region’ here the author is referring to the external genitalia and the anal region. Better to state that clearly – the current wording is confusing, especially the use of ‘anorectal’.

5. This sentence is not critical in conveying important information and has therefore been deleted.

Would be interesting to examine hh signalling in the GT [via qPCR] to confirm the conclusion that urethral defect is a direct result of disrupted hedgehog signaling in the genital tubercle.

6. We interpret this comment to be very similar to comment #3 above and have responded to both together; therefore, please see response to #3 above.

**Reviewer #3:**

This is to elucidate function of GLI3 during testicular development in mice. Using Gli3XtJ mutant mouse line, spontaneous and semi-dominant mutation, the authors find reduction of Leydig cells in the mutants leading to decline of testicular hormones. Androgen supplementation partially rescues some of abnormalities such as delay/lack of testicular descent but not hypospadias. Based on those results, the authors propose a novel function of activated form of GLI3 in Leydig cells as a regulator of male hormone productions.

This is a potentially interesting story to uncover new mechanisms for development of the male reproductive system. Data presented in each figure well support the authors’ conclusions. However, the analyses are superficial and observational for the current format. This reviewer would suggest some additional attempts to understand mechanistic features of the abnormalities found in the mutant mice.

1. At the several places including the author summary, the authors state there is an interaction between the Hedgehog and androgen signaling pathways. What are the data to support this argument? Data shown in Fig 2B suggest that the production of testosterone is regulated by Hedgehog signaling activity (or other GLI3 activities), which is different from the idea of interaction.

This is an important insight; we have significantly altered our discussion section and included a new model figure (new Figure 8) to improve clarity in our thought process that lead to our conclusion that androgen and HH signaling pathways interact in male sex differentiation (see page 14, Line 320). Briefly, it is true that we show that HH regulates androgen production in the fetal Leydig cell; however, our intention was an overarching concept that these pathways work together to promote the overall male sex phenotype development and differentiation. For example, androgen binding to AR in external genitalia stimulates Indian (and Sonic) hedgehog ligand production and therefore downstream signaling. Further, in prostate, the epistatic nature is not yet clear, but the two pathways have been shown to be intertwined.

2. For Fig 1J, it would be helpful to show gross photos to indicate how measurements were done to get ADG. Please provide information which stage(s) the authors made this observation (seemingly at E16.5, but description could be much clearer). Did the authors check genders? If so, how? By checking structures of the reproductive tracts? Presence/absence of Sry? Are there any possibilities that the Gil3XtJ mutation causes sex reversal?

We apologize for the lack of clarity in these methods. The figure below shows representative images of how we measured AGD (for reviewers only). We included additional language within the methods section that outlines our method. We also included text to describe how we sexed embryos, which included evaluation of structures within the tracts when possible and PCR for Sry when we did not have access to tracts. Please see page 19, starting Line 468 within the Materials and Methods section. We have no indication that the *Gli3^XtJ^* mutation causes sex reversal as our data indicate that besides fewer dysfunctional fetal Leydig cells, the testis develops in normal fashion (Figure 4).

3. What is known about the mechanisms of how transcription of Insl3 and/or production of testosterone are regulated by Hedgehog signaling or other functions of activated GLI3? Is there any information to speculate this is a direct interaction or secondary? Data shown in Fig. 2AB suggest that GLI3 is not an essential component for production of testicular hormones, but plays a role to augment productions. What is known about the mechanisms of stage-dependent massive increases of testicular hormones and what is the authors’ speculation how GLI3 would be involved in this mechanism?

This is a very important question and one that is under intense investigation by our lab and others. We have identified putative GLI binding sites in enhancer regions in loci of *Sf1* (including the putative fetal Leydig cell enhancer reported by Shima *et al.* (1)) and other steroidogenic enzyme genes (2); however, transfection analysis in fetal testes (as in Figure 7) and other Leydig cell lines (MA10) have not supported their specificity. Our data suggest that GLI3 contributes to stabilization of the fetal Leydig cell identity likely through indirect means. In addition, we posit that it cooperates with other yet unknown factors to promote differentiation and maturation of a critical mass of fetal Leydig cells to produce sufficient androgens to promote differentiation of all downstream male phenotypes. We included language to this effect in the discussion starting on page 17, Line 416.

(1) Shima Y, Miyabayashi K, Baba T, OtakeH, Oka S, Zubair M, Morohashi K. Identification of an Enhancer in the Ad4BP/SF-1 gene specific for fetal Leydig cells. Endocrinology. 2012;153: 417-25.
(2) Tang C, Pan Y, Luo H, Xiong W, Zhu H, Ruan H, et al. Hedgehog signaling stimulates the conversion of cholesterol to steroids. Cell Signal. 2015;27(3):487-97.

4. Data shown in Fig 3 are intriguing. They convincingly depict different behaviors of cranial suspensory ligament (CSL), which is testosterone-dependent, and caudal gubernaculum ligament, which is not. Similar to above (#3), what is known about the mechanisms of regression of CSL and how/which step exogenous DHT would rescue persistence of fibers in the ligament?

The essential role of androgens in the regression of the CSL comes from animal studies that show that disturbances in androgen synthesis or action result in retained CSL. Examples include mice lacking the AR or rodents treated with AR antagonist, flutamide; mice treated with estrogen; and rats exposed to phthalates, which interferes with fetal Leydig cell differentiation and androgen synthesis. Similarly, female rodents exposed to androgens result in the descent of the ovary further supporting the role of androgens in the regression of CSL. Although it is reported that the CSL is a fibromuscular structure composed of smooth muscle fibers and abundant collagen, there are few details regarding the collagen content of the CSL. Instead, much of the focus has been placed on the development of the gubernaculum, which requires INSL3 for maturation. To clarify and highlight this interesting finding, we have included more information in the discussion section (starting page 16, Line 393). To our knowledge, very few studies have administered exogenous DHT to rescue failure of CSL regression (Hammes et al 2005 Cell); this report will be the first to highlight the impact of DHT on collagen fibers in the CSL. Our studies show that DHT was able to reduce the collagen content of the CSL; however, transabdominal descent also requires INSL3 and hence DHT administration was not able to rescue the descent completely.

5. It is reported that increased levels of MIS can cause failure of testicular descent. Although it is not quantitative, the image shown in Fig 4H&I may prompt to speculate one of the possible reasons for the delay of testicular descent is over produced MIS.

Although AMH (MIS) was initially proposed to be involved in the transabdominal phase of testicular descent (1), there was no direct evidence to prove this hypothesis as *Amh^-/-^* mice demonstrated normal testis descent (2, 3). Szczepny et al have shown that cyclopamine treatment increases *Amh* mRNA, but it was inconclusive with an n=1 (4).

(1) Josso N, Fekete C, Cachin O, Nezelof C, Rappaport R. Persistence of Müllerian ducts in male pseudohermaphroditism and its relationship to cryptorchidism. Clin Endocrinol (Oxf). 1983; 19: 247–258.
(2) Behringer RR, Finegold MJ, Cate RL. Müllerian-inhibiting substance function during mammalian sexual development. Cell (1994) 79(3):415–25
(3) Bartlett JE, Lee SM, Mishina Y, et al. Gubernacular development in Müllerian inhibiting substance receptor-deficient mice. BJU Int. 2002;89:113–118
(4) Szczepny, A., Hogarth, C. A., Young, J., & Loveland, K. L. (2009). Identification of Hedgehog Signaling Outcomes in Mouse Testis Development Using a Hanging Drop-Culture System. Biology of Reproduction, 80 (2), 258 – 263

We evaluated *Amh (Mis)* and another Sertoli cell marker, *Sox9*, to assess the effect of *Gli3* loss on Sertoli cell development and function. The expression levels of both *Amh* and *Sox9* were unaffected by the *Gli3^XtJ^* mutation as shown in the adjacent figure.
